# Conflicts of interest and industry funding declared in systematic reviews of interventions for six common diagnoses

**DOI:** 10.1080/02813432.2025.2519660

**Published:** 2025-06-27

**Authors:** Marek Czajkowski, Louise Olsson

**Affiliations:** a School of Medical Sciences, Örebro University, Örebro, Sweden; b Avestahälsan Medical Centre, Avesta, Sweden; c Centre for Assessment of Medical Technology, Örebro University Hospital, Örebro, Sweden

**Keywords:** Conflicts of interest, disclosure, systematic reviews, chronic disease, primary health care

## Abstract

**Background:**

There is a lack of data on the prevalence of conflicts of interest (COI) declared in systematic reviews over time.

**Methods:**

PubMed was searched for systematic reviews on interventions for chronic obstructive pulmonary disease, type 2 diabetes mellitus, hypertension, dementia, major depression, and osteoarthritis from 2010 and 2019. Selection was conducted by two independent authors, with disagreements resolved in consensus. COI and funding disclosures were extracted. COI were categorised using a specific framework.

**Results:**

746 systematic reviews were included. One third involved pharmacological interventions. Systematic reviews from China increased from 4% to 21% between 2010 and 2019; Cochrane reviews decreased from 19% to 4%.

Systematic reviews presenting a COI statement increased from 79% to 94%. Those with at least one author declaring individual financial COI decreased from 22% to 17% but remained at 22–23% when excluding systematic reviews from China. Almost 1 in 3 systematic reviews on pharmacological interventions and invasive procedures declared individual financial COI for 2019. Individual intellectual COI were declared in 2.5% and other types of COI were very rare.

Systematic reviews presenting a funding statement increased from 65% to 81%; industry funding decreased from 6% to 3.4%. Adding industry funding to the prevalence of systematic reviews declaring financial COI only made a marginal difference.

**Conclusions:**

The proportion of systematic reviews on interventions for common diagnoses declaring individual financial COI remained consistent at approximately one in five for both 2010 and 2019, underscoring the need for further research into the implications of this finding.

## Introduction

Conflicts of interest (COI) among authors of scientific papers suggest that their secondary interests could have influenced their research [1]. As this is true also for systematic reviews, COI declarations provide readers with essential information to better understand the context of published reviews. In biomedical science, the request for transparency on COI was initiated by the International Committee of Medical Journal Editors (ICMJE) in the late 1980s [2], and it was promoted by independent producers of systematic reviews, like the Cochrane Collaboration [3]. Financial COI have traditionally been the focus, but lately there has been increasing interest in other types of COI, e.g. intellectual COI. Detailed guidance on the extended disclosure of COI and funding sources is now included in the latest updates of the reporting guidelines for systematic reviews (PRISMA 2020) [4], as well as in the COI disclosure forms from ICMJE [5].

Systematic reviews on the effectiveness of interventions are crucial, as they inform clinical practice, guidelines, and health policies [6]. Systematic reviews investigating interventions for common diagnoses are expected to have an even greater impact due to the larger patient populations they concern. Given the position of systematic reviews at the top of the hierarchy of evidence, it is vital to thoroughly understand any potential effect of COI among their authors. This includes all aspects of a systematics review, such as planning, conduct, analysis, reporting, and conclusions [7,8]. Cochrane introduced restrictions on authorship for financial COI in 2020, such as disallowing authorship by current or past industry employees [9]. However, the literature on the prevalence of COI in systematic reviews remains sparse, with only a few cross-sectional studies on COI statements and their content in substantial samples of systematic reviews. Indeed, Page et al. [10] investigated a random sample of systematic reviews on therapeutic interventions published in February 2014 and found that 148/164 (90%) had a COI statement. Hakoum et al. [11] found that 194/200 (97%) of similar systematic reviews (100 published in Core Clinical Journals in 2015 and 100 published by Cochrane) included a COI statement, and 60 (30%) had at least one author with financial COI. Nguyen et al. [12] also reported that 276/294 (94%) of systematic reviews on health interventions published in November 2020 had a COI statement, and 29 (11%) had positive COI declarations.

However, we could not find any studies investigating changes in the prevalence of COI statements, or more specifically, the content of these declarations, in systematic reviews focused on interventions for common diagnoses over time. Industry funding of systematic reviews on interventions was found to be uncommon in the cross-sectional studies mentioned: 4/164 (2.4%) in Page et al. 3/200 (1.5%) in Hakoum et al. and 6/294 (2%) in Nguyen et al. [10–12]. Yet, the prevalence of industry funding in addition to financial COI among authors of systematic reviews has not been comprehensively examined.

The primary aim of this study was therefore to determine the prevalence of financial and other types of COI declared in systematic reviews on interventions for six common diagnoses from two distinct years, with an intermediate period to investigate changes over time. A secondary aim was to assess the prevalence of declared funding from industry and any association with the declarations of financial COI.

## Material and methods

### Study design

A cross-sectional study on COI and funding statements of systematic reviews from 2010 and 2019 was designed. The year 2010 was the first year after the publication of the PRISMA reporting guidelines in 2009 [13], and 2019 was the last representative year before the disruption of the Covid-19 pandemic, as we did not know how the pandemic would affect the production of systematic reviews while planning this study. The scope of the study was limited to systematic reviews of interventions for diagnoses with a prevalence of at least 1% of the adult population, assuming these systematic reviews have a greater impact on healthcare than those focused on less common diagnoses. In order to keep the inclusion of systematic reviews feasible, six arbitrary diagnoses were chosen: type 2 diabetes mellitus (T2DM), chronic obstructive pulmonary disease (COPD), essential hypertension, dementia of Alzheimer’s or vascular type, major depressive disorder, and osteoarthritis.

### Literature search

A medical information specialist at the Medical Library, Örebro University conducted the literature search based on the following MeSH terms: ‘Diabetes Mellitus, Type 2’, ‘Pulmonary Disease, Chronic Obstructive’, ‘Hypertension’, ‘Dementia’, ‘Depressive Disorder, Major’ or ‘Osteoarthritis’, and ‘Systematic review’ as title or publication type. The search was restricted to PubMed (MEDLINE) without any filters. The search periods were limited to January 1 to December 31, 2010, and January 1 to December 31, 2019. The search was conducted on the 6^th^ of July 2021. The complete search strategy is presented in the Supplementary Table S1.

### Inclusion criteria

The following pre-defined criteria were adopted to include publications

- described as systematic reviews or meta-analyses based on a systematic literature search, or if the authors referred to the PRISMA reporting guidelines [13],
- investigating interventions for any of the six chosen diagnoses,
- on adult patients,
- including primary studies using quantitative data,
- focused on clinically relevant outcomes such as symptoms, mortality, and harms. Commonly used parameters and laboratory data for disease management e.g. blood pressure and HbA1c in systematic reviews on hypertension and T2DM, respectively, were also accepted.
- with a full-text version available in English,
- not retracted according to PubMed.

Two authors (MC, LO) initially screened titles and abstracts independently. There was substantial agreement between the two authors’ judgements (κ = .669) [14]. Remaining discrepancies were resolved in consensus for inclusion in the study. The full-text versions of the included publications were then retrieved.

### Data extraction

Basic characteristics (diagnosis, type of intervention, country, journal) were extracted by one author (MC). During this phase, if ineligibility was detected in the full texts, the publication was discussed among the authors, prior to exclusion. Systematic reviews on two, or more, of the chosen diagnoses were classified according to the largest number of included primary studies, or, if equal, by the diagnosis with the largest number of participants. The investigated interventions were categorized as follows: 1) pharmacological, 2) invasive procedures, including surgery and interventional radiology, 3) psychological, social and nursing, 4) physiotherapeutic and occupational therapy, 5) dietary, including supplements and vitamins, 6) alternative and complementary treatments, 7) systematic reviews investigating two or more different types of interventions (multiple interventions). The specified publication years of systematic reviews, 2010 or 2019, were based on the search periods and registration of the systematic review in PubMed. In most cases, this was the same as the actual publication year in the journals, with a few exceptions. The first affiliation of the first author determined the country of origin of the systematic review. The five most prevalent countries were reported separately, and the remaining were referred to as ‘other countries’. The publishing journals were dichotomized into ‘Cochrane’ for Cochrane Database of Systemic Reviews and ‘other journals’.

Data on COI and funding were extracted as reported by the authors, and variables were coded binary (yes/no) for each systematic review by one author (MC). No attempt was made to assess any quantitative aspects of the COI or funding disclosed by the authors, nor was the validity of these statements questioned.

Data extraction on COI involved the presence/absence of a COI statement, and secondly, whether this statement declared (i.e. positive declaration) or denied any COI. Positive COI declarations were further subcategorized according to a published framework [11] that distinguishes between COI at the individual and institutional levels. Our interpretation and use of this framework is outlined in Supplementary Table S2. Individual COI concerned relationships established by the author(s), whereas institutional COI were about relationships established by the author′s institution. Individual COIs were divided into financial, professional (non-financial, related to the author’s clinical activities, e.g. including the intervention investigated in the systematic review), and intellectual (non-financial, related to the author’s scholarly activities, e.g. previous research on the same topic). Institutional COIs were divided into financial (benefiting the author’s affiliated institution, e.g. grants from the industry to the institution) and advocatory (non-financial, related to the author’s affiliated institution’s mission, e.g. an institution with a strong position on the topic investigated in the systematic review). The threshold for a positive classification of a systematic review was having at least one author declaring any specific type of COI. Several reasons for the same type of COI declared by one or more authors were summarised as one specific type of COI per systematic review. If the COI statement provided declarations of more than one type of COI, all the different types were assigned to that systematic review.

Data extraction on funding involved the presence/absence of a funding statement, and secondly, whether the statement declared or denied any funding. Declared funding sources were further dichotomized into for-profit organisations (labelled ‘industry’), or other organisations. Names of organisations that were unfamiliar to the authors of this study, were searched online to minimize the risk of misclassification. The classifications of individual financial COI and industry funding in two randomly selected samples − 20% (*n* = 150), and 10% (*n* = 75) – of the systematic reviews were cross-checked (see Acknowledgements and LO, respectively), with minor corrections. In addition, all systematic reviews categorised with non-financial COI (*n* = 27) and industry funding (*n* = 49) were double-checked (LO). Disagreements were discussed until consensus was reached, and adjustments were made accordingly.

### Statistics

Descriptive statistics and stratified frequency tables were used to present the data. The primary findings – proportions of systematic reviews declaring COI, denying COI and having no COI statements – were presented as percentages of all the included systematic reviews for that year. Systematic reviews disclosing funding from industry were cross tabulated with those disclosing individual financial COI to identify any overlap. IBM SPSS Statistics, version 28.0.0.0 (190), was used. Data, beyond the supplementary material, is available upon request.

## Results

**Figure 1.**
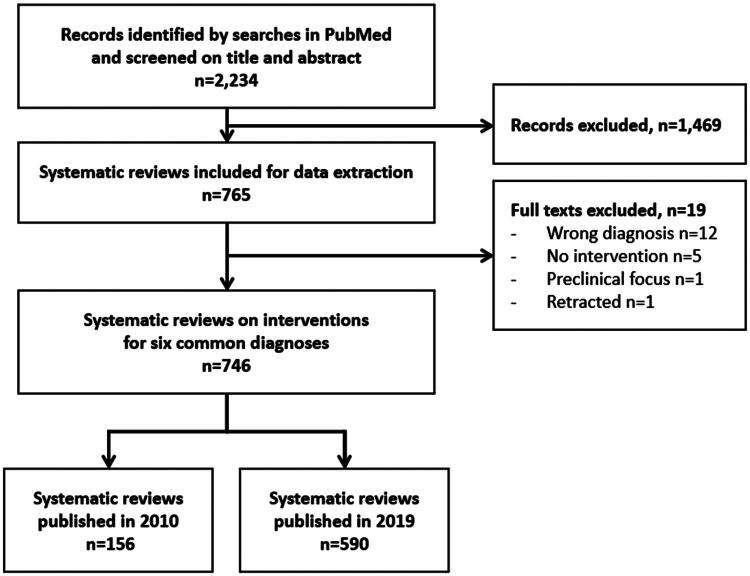
Study flow diagram.

The search strategy identified 2,234 records. The screening process found 765 relevant systematic reviews but 19 of them were judged ineligible on the full-text level (Figure 1). In all, 156 systematic reviews for 2010 and 590 for 2019 were included, an increase of 278%.

Basic characteristics of the included systematic reviews are presented in Table 1. Systematic reviews on interventions for T2DM and osteoarthritis increased the most between 2010 and 2019 and accounted for nearly half of all included systematic reviews for 2019. Pharmacological interventions were most common, constituting one third of systematic reviews for both 2010 and 2019. The number of systematic reviews from China was 6 (4%) for 2010 and 124 (21%) for 2019. The proportion of systematic reviews published by Cochrane for those years were 19% and 4%, respectively.

**Table 1.** Basic characteristics of the included systematic reviews for 2010 and 2019. Numbers in parentheses are column percentages.

Characteristics	Systematic reviews 2010 *n* = 156	Systematic reviews 2019 *n* = 590
Diagnosis		
T2DM	25 (16)	161 (27)
COPD	28 (18)	61 (10)
Hypertension	29 (19)	77 (13)
Dementia	16 (10)	83 (14)
Depression	35 (22)	84 (14)
Osteoarthritis	23 (15)	124 (21)
Intervention		
Pharmacological	50 (32)	189 (32)
Invasive procedures	15 (10)	86 (15)
Psychological, social, nursing	38 (24)	101 (17)
Physio-, occupational therapy	23 (15)	93 (16)
Dietary	20 (13)	70 (12)
Alternative, complementary	3 (2)	22 (4)
Multiple interventions	7 (4)	29 (5)
Country		
China	6 (4)	124 (21)
UK	34 (22)	69 (12)
USA	23 (15)	71 (12)
Australia	17 (11)	52 (9)
Canada	17 (11)	32 (5)
Other countries	59 (38)	242 (41)
Journal		
Cochrane	29 (19)	22 (4)
Other journals	127 (81)	568 (96)

T2DM - type 2 diabetes mellitus, COPD- chronic obstructive pulmonary disease.

### Conflicts of interest and funding

The proportion of systematic reviews with a statement on COI was 79% for 2010 and 94% for 2019 (Table 2). The presence of a COI statement by basic characteristics of the included systematic reviews is detailed in Supplementary Table S3.

**Table 2.** Conflicts of interest (COI) and industry funding as declared in the included systematic reviews for 2010 and 2019.

Characteristics	Systematic reviews 2010 *n* = 156	Systematic reviews 2019 *n* = 590
COI statement		
Disclosed	123 (79)	552 (94)
Missing	33 (21)	38 (6)
Declarations in COI statement		
Any COI	39 (25)	121 (21)
Individual financial COI*	35 (22)	103 (17)
Individual intellectual COI*	6 (4)	15 (2.5)
Individual professional COI*	1 (0.6)	2 (0.3)
Institutional financial COI*	0	2 (0.3)
Institutional advocatory COI*	0	2 (0.3)
Authors declare they had no COI	84 (54)	431 (73)
Funding statement		
Disclosed	101 (65)	475 (81)
Missing	55 (35)	115 (19)
Declarations in funding statement		
Any funding	87 (56)	308 (52)
Industry funding	10 (6)	20 (3.4)
Authors declare there was no funding	14 (9)	167 (28)

*More than one type of COI could be declared per systematic review. In total six systematic reviews declared individual financial COI in addition to other types of COI (two for 2010 and four for 2019), see Supplementary Table S4.

Numbers in parentheses are column percentages, and the denominator is the total number of systematic reviews for 2010 and 2019, respectively.

The presence of any COI was declared in 25% of systematic reviews for 2010 and in 21% for 2019. For 2010, 54% of all systematic reviews declared no COI, and this proportion was 73% for 2019. Individual financial COI were declared in 35/156 (22%) systematic reviews for 2010 and in 103/590 (17%) for 2019.

Other types of COI beyond the individual financial type are presented in Supplementary Table S4. In all, 28 cases of COI of other types than individual financial COI were declared in 27 systematic reviews, most of them for 2019. Nine (33%) of the systematic reviews were endorsed by Cochrane, while the remaining 18 (67%) were published in other journals. Intellectual COI were the most common type of non-financial COI (Supplementary Table S6), whereas other types were very rare. In three cases the declared COI did not fit into the chosen framework.

The proportion of systematic reviews with a funding statement was 65% for 2010 and 81% for 2019 (Table 2). For 2010, a funding source was declared in 56% of the systematic reviews compared to 52% for 2019. Funding from industry was declared in 6% of systematic reviews for 2010, and in 3% for 2019. The presence of a statement on funding and the presence of funding from industry by basic characteristics of the included systematic reviews are detailed in Supplementary Tables S7 and S8.

### Financial conflicts of interest

The proportions of systematic reviews declaring individual financial COI by diagnosis varied widely, between 4% and 31% for 2019, and were generally lower than those for 2010, except for osteoarthritis (Table 3). For 2019, 31% of systematic reviews on pharmacological interventions and 29% of those on invasive procedures declared individual financial COI, compared with 42% and 20% for 2010, respectively. Individual financial COI were declared in 50% of systematic reviews from Cochrane for 2019, compared to 16% in other journals. Systematic reviews from China declared no individual financial COI among the authors for either 2010 or 2019. If systematic reviews from China were excluded from the analysis, the proportion of systematic reviews declaring individual financial COI was 22% (103/466) for 2019 and 23% (35/150) for 2010 (Supplementary Table S5).

**Table 3.** Individual financial conflicts of interest (COI) as declared in the included systematic reviews (SRs) for 2019 and 2010 by basic characteristics. Numbers in parentheses are percentages of the row total.

Characteristics	SRs with ≥ 1 author declaring individual financial COI	SRs by authors declaring no COI, or only other than the individual financial type of COI	Missing information (no COI statement)	Total
Systematic reviews 2019				
Diagnosis				
T2DM	26 (16)	127 (79)	8 (5)	161
COPD	11 (18)	45 (74)	5 (8)	61
Hypertension	3 (4)	72 (94)	2 (3)	77
Dementia	7 (8)	70 (84)	6 (7)	83
Depression	26 (31)	50 (60)	8 (10)	84
Osteoarthritis	30 (24)	85 (69)	9 (7)	124
Intervention				
Pharmacological	58 (31)	122 (65)	9 (5)	189
Invasive procedures	25 (29)	55 (64)	6 (7)	86
Psychological, social, nursing	4 (4)	86 (85)	11 (11)	101
Physio-, occupational therapy	3 (3)	84 (90)	6 (6)	93
Dietary	8 [11]	61 (87)	1 (1)	70
Alternative, complementary	0	19 (86)	3 (14)	22
Multiple interventions	5 (17)	22 (76)	2 (7)	29
Country				
China	0	119 (96)	5 (4)	124
UK	18 (26)	48 (70)	3 (4)	69
USA	19 (27)	44 (62)	8 (11)	71
Australia	10 (19)	39 (75)	3 (6)	52
Canada	12 (38)	19 (59)	1 (3)	32
Other countries	44 (18)	180 (74)	18 (7)	242
Journal				
Cochrane	11 (50)	11 (50)	0	22
Other journals	92 (16)	438 (77)	38 (7)	568
Total for 2019	103 (17)	449* (76)	38 (6)	590
Systematic reviews 2010				
Diagnosis				
T2DM	5 (20)	17 (68)	3 (12)	25
COPD	8 (29)	16 (57)	4 (14)	28
Hypertension	4 (14)	21 (72)	4 (14)	29
Dementia	4 (25)	6 (38)	6 (38)	16
Depression	11 (31)	18 (51)	6 (17)	35
Osteoarthritis	3 (13)	10 (43)	10 (43)	23
Intervention				
Pharmacological	21 (42)	21 (42)	8 (16)	50
Invasive procedures	3 (20)	6 (40)	6 (40)	15
Psychological, social, nursing	6 (16)	28 (74)	4 (10)	38
Physio-, occupational therapy	2 (9)	13 (56)	8 (35)	23
Dietary	2 (10)	12 (60)	6 (30)	20
Alternative, complementary	0	3 (100)	0	3
Multiple interventions	1 (14)	4 (57)	2 (29)	7
Country				
China	0	4 (67)	2 (33)	6
UK	6 (18)	23 (68)	5 (15)	34
USA	7 (30)	10 (43)	6 (26)	23
Australia	3 (18)	9 (53)	5 (29)	17
Canada	6 (35)	9 (53)	2 (12)	17
Other countries	13 (22)	33 (56)	13 (22)	59
Journal				
Cochrane	7 (24)	21 (72)	1 (3)	29
Other journals	28 (22)	67 (53)	32 (25)	127
Total for 2010	35 (22)	88** (56)	33 (21)	156

*Authors declare they have no COI (*n* = 431) or declare they have COI, but it is not individual financial COI (*n* = 121-103 = 18): 431 + 18 = 449.

**Authors declare they have no COI (*n* = 84) or declare they have COI, but it is not individual financial COI (*n* = 39-35 = 4): 84 + 4 = 88.

### Funding from industry vs individual financial COI

Any overlap between declarations of funding from industry and individual financial COI in the included systematic reviews is presented in Table 4. For 2019, 20/590 (3.4%) systematic reviews declared funding from industry; 15/590 (2.5%) declared both individual financial COI and funding from industry, and 5/590 (0.8%) declared funding from industry, but no individual financial COI. For 2010, 10/156 (6.4%) systematic reviews declared funding from industry; 8/156 (5.1%) declared both individual financial COI and funding from industry, and 2 (1.3%) declared funding from industry, but no individual financial COI.

**Table 4.** Funding from industry and individual financial conflicts of interest (COI) as declared by authors of the included systematic reviews (SRs) for 2019 and 2010.

		Individual financial conflicts of interest			
		SRs with ≥ 1 author declaring individual financial COI	SRs by authors declaring no COI, or only other than individual financial COI	SRs missing information (no COI statement)	Total
Funding from industry	Systematic reviews 2019				
	SRs declaring funding from industry	15 (2.5)	5 (0.8)	0	20 (3.4)
	SRs declaring no funding from industry or only non-industry funding	72 (12.2)	366 (62)	17 (2.9)	455 (77.1)
	SRs missing information (no funding statement)	16 (2.7)	78 (13.2)	21 (3.6)	115 (19.5)
	Total for 2019	103 (17.5)	449 (76.1)	38 (6.4)	590 (100)
	Systematic reviews 2010				
	SRs declaring funding from industry	8 (5.1)	2 (1.3)	0	10 (6.4)
	SRs declaring no funding from industry or only non-industry funding	16 (10.3)	62 (39.7)	13 (8.3)	91 (58.3)
	SRs missing information (no funding statement)	11 (7.1)	24 (15.4)	20 (12.8)	55 (35.3)
	Total for 2010	34 (21.8)	87 (55.8)	35 (22.4)	156 (100)

Numbers in parentheses are percentages of the total number of systematic reviews investigated in 2019 and 2010, respectably.

## Discussion

Individual financial COI were declared in 22% and 17% of systematic reviews on interventions for six common diagnoses for 2010 and 2019, respectively. The share of systematic reviews from China increased from 4% to 21% between the investigated years. When these were excluded, the proportion of systematic reviews with authors declaring individual financial COI remained at 22-23% for both years. Almost one third of systematic reviews on pharmacological interventions and invasive procedures declared individual financial COI in 2019, while intellectual COI were declared in 2.5%, and other types of COI even more rarely. Funding from industry was declared in less than 5% of the included systematic reviews and mostly, though not completely, overlapped with declarations of individual financial COI.

There is little data on the prevalence of different types of COI in systematic reviews on interventions for the same, common diagnoses as investigated in the present study, but at least one study by Lieb et al. [15] reported a somewhat similar result. They found that 25/95 (26%) systematic reviews published in 2010-2013 on psychological therapies for adults with anxiety, personality disorders and/or major depressive disorders declared any type of COI, and that these were financial in 22 (23%) cases, and non-financial in 5 (5%). Heigle et al. [16] on the other hand found that merely 3/53 (6%) of systematic reviews on interventions for knee osteoarthritis published in 2016-2019 declared financial COI among at least one of the authors, but that another 9 (17%) had authors with undisclosed financial COI that were identified *via* external sources.

Investigations of COI declared in systematic reviews on interventions for other less frequent diagnoses are also limited to a few studies. Roundtree et al. [17] found that 11/26 (42%) of systematic reviews on biologic therapies for rheumatoid arthritis published up to 2005 declared financial COI. Dunn et al. [18] found that 7/26 (27%) systematic reviews on the use of neuraminidase inhibitors for influenza published in 2005-2014 declared financial COI. Crow et al. [19] found that in 4/12 (33%) systematic reviews on pharmacological treatment of alcohol disorders published 2016-2019, the authors declared financial COI.

In the present study, we compared the prevalence of various types of COI in systematic reviews on interventions for the same diagnoses from two different calendar years. We have not been able to identify any other study with a similar objective. However, COI declared in systematic reviews on psoriasis have been investigated in two separate studies. Gómez-García et al. [20] analysed 220 systematic reviews published until 2016 on psoriasis, including 129 (59%) focused on treatment, and found that 135 (61%) declared COI. Later, Kee et al. [21] found that 22/27 (82%) systematic reviews on the treatment of psoriasis, published 2016-2020, had at least one author with COI. The presence of COI’s therefore appears to be markedly higher in systematic reviews on psoriasis than in systematic reviews on the diagnoses that are included in our study.

All these findings highlight financial COI as the most commonly declared type of COI in systematic reviews, even though the prevalence may vary across different topics. The inclusion criteria for the types of systematic reviews investigated, differences in interventions used for specific diseases, and industry interests in particular fields obviously influence that prevalence. For specific, narrow topics, the prevalence of COI could even be inflated by a single researcher declaring COI in multiple publications, while other systematic reviews focus on interventions with minimal exposure to financial interest, such as alternative or complementary interventions.

Hakoum et al. found that intellectual COI were declared in 24/200 (12%) systematic reviews on interventions published in 2015, and professional COI in 2.5% (5/200). These proportions are higher than in our study, with 2.5% and 0.3%, respectively, for the systematic reviews for 2019. The aforementioned study by Lieb et al. [15] found that non-financial COI were declared in 4.2% of the included systematic reviews. This low reporting rate of other types of COI, beyond the individual financial type, may be due to the absence of widely accepted definitions, or clear guidance [5]. We adopted a framework for the categorization of COI in systematic reviews proposed in 2016 (11) but found it difficult to categorize all declared COI, such as financial ties of individuals or institutions with non-profit organizations, or for-profit companies that were not manufacturers of a drug or device. An updated operational definition and a new framework for categorizing secondary interests in health research, along with further guidance for categorizing non-financial or institutional COI, have recently been published [22].

The prevalence of funding from industry in this study was 3.4% for systematic reviews from 2019. This is similar to other cross-sectional studies on random samples of systematic reviews [10–12]. For specific diagnoses, such as psoriasis, Gómez-García et al. [20] found that 61/220 (28%) of systematic reviews published until 2016 were funded by industry, 79% by one pharmaceutical company. Furthermore, meta-analyses without a systematic review component may be funded by industry more often. For instance, Ebrahim et al. [23] found that 46/185 (25%) meta-analyses on antidepressants for depression published 2007-2014 were sponsored by the manufacturer of the drug.

Transparency in COI and funding statements is recommended by medical journal editors to ensure honest dissemination of research findings and provide readers with an opportunity to assess any influence of COI [5]. While this is an important step, it is also worth remembering that merely declaring COI does not eliminate the risks they pose [24]. The potential for biased decisions throughout the research process still exists in systematic reviews, despite transparent disclosures. According to psychological research, readers tend to underestimate possible bias caused by COI, even when they are informed about them [25]. Meanwhile, systematic reviews that declare financial COI or industry funding have been found to report more favourable conclusions compared to reviews that deny such financial relationships [7]. This underlines the importance for further research on this topic.

Strengths of this study include the search strategy built on Mesh-terms in PubMed, and screening for relevant systematic reviews by two independent authors. The selection of studies was initially limited to screening on title and abstracts to ensure that the authors were blinded to COI and funding statements in the full-text versions of the publications. A significant amount of extracted data was double-checked. COI were categorized according to an established framework [11].

There are a few limitations as well. Systematic reviews from the selected years, 2010 and 2019, may not be representative. The manual extraction of data on COI from the systematic reviews was not always straightforward as in addition to the most common location at the end the paper it could be embedded in the title page, method section, funding statement, acknowledgments, as well as in supplementary materials or in the protocol [26]. Despite a meticulous systematic approach, there remains a limited risk that relevant information on COI or funding was missed. The cut-off for a positive COI classification was at least one author declaring COI per systematic review, but more details on the proportion of authors with COI or their roles were not investigated.

In conclusion, individual financial COI are declared in approximately every fifth systematic review on interventions for six common diagnoses. For pharmacological treatments and invasive procedures, COI are declared in close to one third of these important systematic reviews.

## Supplementary Material

Supplemental Material
