# Supplementary material for: Conflicts of interest and industry funding declared in systematic reviews of interventions for six common diagnoses
Source: Scand J Prim Health Care. 2025 Jun 27;44(1):1–9. doi: 10.1080/02813432.2025.2519660 (PMC12918370; doi:10.1080/02813432.2025.2519660)
Supplement: Supplemental Material [file IPRI_A_2519660_SM7616.docx]

**Supplementary materials**

**Table S1.** Search strategy.

| Database: PubMed (including MEDLINE), no filters, date: 210706 |
| --- |
| **Chronic Obstructive Pulmonary Disease**  (Pulmonary Disease, Chronic Obstructive [MeSH Terms])  (Systematic Review[Title])  (Systematic Review[Publication Type])  2 OR 3  ("2010/01/01"[Date - Publication] : "2010/12/31"[Date - Publication])  ("2019/01/01"[Date - Publication] : "2019/12/31"[Date - Publication])  1 AND 4 AND 5 - **42 results**  1 AND 4 AND 6 - **150 results** |
| **Type 2 Diabetes Mellitus**  (Diabetes Mellitus, Type 2 [MeSH Terms])  (Systematic Review[Title])  (Systematic Review[Publication Type])  2 OR 3  ("2010/01/01"[Date - Publication] : "2010/12/31"[Date - Publication])  ("2019/01/01"[Date - Publication] : "2019/12/31"[Date - Publication])  1 AND 4 AND 5 - **63 results**  1 AND 4 AND 6 - **442 results** |
| **Hypertension**  (Hypertension [MeSH Terms])  (Systematic Review[Title])  (Systematic Review[Publication Type])  2 OR 3  ("2010/01/01"[Date - Publication] : "2010/12/31"[Date - Publication])  ("2019/01/01"[Date - Publication] : "2019/12/31"[Date - Publication])  1 AND 4 AND 5 - **81 results**  1 AND 4 AND 6 - **293 results** |
| **Dementia**  (Dementia [MeSH Terms])  (Systematic Review[Title])  (Systematic Review[Publication Type])  2 OR 3  ("2010/01/01"[Date - Publication] : "2010/12/31"[Date - Publication])  ("2019/01/01"[Date - Publication] : "2019/12/31"[Date - Publication])  1 AND 4 AND 5 - **67 results**  1 AND 4 AND 6 - **403 results** |
| **Depression**  (Depressive Disorder, Major [MeSH Terms])  (Systematic Review[Title])  (Systematic Review[Publication Type])  2 OR 3  ("2010/01/01"[Date - Publication] : "2010/12/31"[Date - Publication])  ("2019/01/01"[Date - Publication] : "2019/12/31"[Date - Publication])  1 AND 4 AND 5 - **81 results**  1 AND 4 AND 6 - **320 results** |
| **Osteoarthritis**  (Osteoarthritis [MeSH Terms])  (Systematic Review[Title])  (Systematic Review[Publication Type])  2 OR 3  ("2010/01/01"[Date - Publication] : "2010/12/31"[Date - Publication])  ("2019/01/01"[Date - Publication] : "2019/12/31"[Date - Publication])  1 AND 4 AND 5 - **59 results**  1 AND 4 AND 6 - **233 results** |
| **All diagnoses (no duplicates)**  2010: 393 results  2019: 1,841 results  **Total: 2,234 results** |

**Table S2.** Framework for classification of conflicts of interest (COI) adopted from Hakoum et al. (11). Headings and subheadings are retrieved from the Hakoum’s paper, but interpretation and examples (in italics) are from of the present paper.

| **Individual COI**   - relationships established by the author | **Institutional COI**   - relationships established by the author’s institution, and the author is aware of it |
| --- | --- |
| **Financial:**   - directly benefiting the author   *Honorarium, consultancy fees, employment, travel reimbursement, patents, research funding.* | **Financial:**   - benefiting the author’s institution   *Financial support to authors´ institution from the manufacturer of a drug or device under consideration for research involving the authors.* |
| **Professional:**   - indirect benefit through clinical activity   *Occupation or professional activities related to the topic of the systematic reviews* | **Advocatory:**   - aligning with author’s institution policy or mission   *Author belongs to institution/organisation that has missions related to the topic of the systematic review.* |
| **Intellectual:**   - indirect benefit through scholarly activity   *Authorship of primary studies included in the systematic review or not included but on similar topic. Editorial function related to the specific issue/publication. Involvement in the development of guidelines.* |  |

**Table S3.** Presence of a statement on conflicts of interest (COI) by basic characteristics in the included systematic reviews for 2019 and 2010. Numbers in parentheses are row percentages.

| **Characteristics** | Systematic reviews reporting statement on COI | Systematic reviews missing statement on COI | **Total** |
| --- | --- | --- | --- |
| **Systematic reviews 2019** |  |  |  |
| **Diagnosis** |  |  |  |
| T2DM | 153 (95) | 8 (5) | **161** |
| COPD | 56 (92) | 5 (8) | **61** |
| Hypertension | 75 (97) | 2 (3) | **77** |
| Dementia | 77 (93) | 6 (7) | **83** |
| Depression | 76 (90) | 8 (10) | **84** |
| Osteoarthritis | 115 (93) | 9 (7) | **124** |
| **Intervention** |  |  |  |
| Pharmacological | 180 (95) | 9 (5) | **189** |
| Invasive procedures | 80 (93) | 6 (7) | **86** |
| Psychological, social, nursing | 90 (89) | 11 (11) | **101** |
| Physio-, occupational therapy | 87 (94) | 6 (6) | **93** |
| Dietary | 69 (99) | 1 (1) | **70** |
| Alternative, complementary | 19 (86) | 3 (14) | **22** |
| Multiple interventions | 27 (93) | 2 (7) | **29** |
| **Country** |  |  |  |
| China | 119 (96) | 5 (4) | **124** |
| UK | 66 (96) | 3 (4) | **69** |
| USA | 63 (89) | 8 (11) | **71** |
| Australia | 49 (94) | 3 (6) | **52** |
| Canada | 31 (97) | 1 (3) | **32** |
| Other countries | 224 (93) | 18 (7) | **242** |
| **Journal** |  |  |  |
| Cochrane | 22 (100) | 0 | **22** |
| Other journals | 530 (93) | 38 (7) | **568** |
| **Total for 2019** | **552 (94)** | **38 (6)** | **590** |
| **Systematic reviews 2010** |  |  |  |
| **Diagnosis** |  |  |  |
| T2DM | 22 (88) | 3 (12) | **25** |
| COPD | 24 (86) | 4 (14) | **28** |
| Hypertension | 25 (86) | 4 (14) | **29** |
| Dementia | 10 (63) | 6 (38) | **16** |
| Depression | 29 (83) | 6 (17) | **35** |
| Osteoarthritis | 13 (57) | 10 (43) | **23** |
| **Intervention** |  |  |  |
| Pharmacological | 42 (84) | 9 (16) | **50** |
| Invasive procedures | 9 (60) | 6 (40) | **15** |
| Psychological, social, nursing | 34 (89) | 4 (11) | **38** |
| Physio-, occupational therapy | 16 (70) | 7 (30) | **23** |
| Dietary | 14 (70) | 6 (30) | **20** |
| Alternative, complementary | 3 (100) | 0 | **3** |
| Multiple interventions | 5 (71) | 2 (29) | **7** |
| **Country** |  |  |  |
| China | 4 (67) | 2 (33) | **6** |
| UK | 29 (85) | 5 (15) | **34** |
| USA | 17 (74) | 6 (26) | **23** |
| Australia | 12 (71) | 5 (29) | **17** |
| Canada | 15 (88) | 2 (12) | **17** |
| Other countries | 46 (78) | 13 (22) | **59** |
| **Journal** |  |  |  |
| Cochrane | 28 (97) | 1 (3) | **29** |
| Other journals | 95 (75) | 32 (25) | **127** |
| **Total for 2010** | **123 (79)** | **33 (21)** | **156** |

**Table S4.** Other types of conflicts of interest (COI), beyond individual financial COI, as declared in the included systematic reviews (n=27) and classified according to the framework by Hakoum (11).

| **Author, Year, Journal** | **Abstracted declarations** | **Type of COI** |
| --- | --- | --- |
| Barrow, 2019, BMC Muscul skel disorders | One reviewer was on the editorial board of the publishing journal | Intellectual |
| Bennet, 2019, BMJ Open | Four reviewers were authors of trials included in the systematic review | Intellectual |
| Bricca, 2019, Am College of Rheumatology | One reviewer was the developer of the Knee Injury and Osteo-arthritis Outcome Score (KOOS) and several other freely available patient-reported outcome measures. This is a questionnaire designed to assess short and long-term patient-relevant outcomes following knee injury. | Intellectual |
| Brown, 2019, BMJ | All authors had financial support via the University of East Anglia from the World Health Organization for the submitted work.  Two reviewers were funded to attend WHO meetings and present review results. | ***Not classified according to framework*** |
| Brown, 2019, Cochrane | One reviewer lead and had responsibility for Cochrane Common Mental Disorders that supported parts of the review process and is largely funded by a grant from the National Institute of Health and Research (NIHR) in the UK.  One reviewer was Chief Investigator of the Community Pharmacy Mood Intervention Feasibility and Pilot Study funded by the National Institute for Health Research. | Intellectual |
| Cole, 2019, J Hum Hypertension | One reviewer was a member of Consensus Action on Salt & Health (CASH) and World Action on Salt & Health (WASH). Both CASH and WASH are non-profit charitable organisations.  One reviewer was Chairman of Blood Pressure UK (BPUK), Chairman of CASH, WASH and Action on Sugar (AoS). BPUK, CASH, WASH and AoS are non-profit charitable organisations. | Institutional advocatory |
| Jones, 2019, Cochrane | One of the reviewers was employed by the university, as an Associate Editor/Deputy Managing Editor with Cochrane Pregnancy and Childbirth. The employment was supported by the National Institute for Health Research, via Cochrane Infrastructure funding to Cochrane Pregnancy and Childbirth. The reviewer had no involvement with the editorial processes for this review update. | Intellectual |
| Leopoldino, 2019, Cochrane | Six reviewers conducted a systematic review that investigated the benefits of the same analgesics for patients with the same diagnosis (+spinal pain) published in the BMJ). Two reviewers were investigators of trial on the same analgesics for another diagnosis (low back pain)  One reviewer was a consultant for Flexion, Merck Serono, and Nestlé | Intellectual  Individual financial |
| MC Elheny, 2019, Sports Health | - One of the reviewers was an associate editor of the publishing   journal | Intellectual |
| Machmutow, 2019, Cochrane | Three reviewers coordinated a guideline update.  Six reviewers participated in publicly funded investigator-initiated primary studies and systematic reviews of interventions for the investigated diagnosis.  Six reviewers had formal training in one the investigated methods.  The expert association DGPPN (German Association for Psychiatry, Psychotherapy and Psychosomatics) (editor of the S3 Guideline) provided financial support for the preparation of the Guideline Update to the coordinators' institution (Department of Medical Psychology, University Medical Center Hamburg-Eppendorf). | Intellectual  Professional  Institutional financial |
| Mallery, 2019, BMC Geriatrics | - Two reviewers were co-founders of the PATH program. They were working to better understand the system implications for larger scale implementation of the program. | Intellectual |
| Maula, 2019, Diab Med | - One reviewer was Chair of the NICE PH38 Guidelines on Early Detection and Prevention of Diabetes. - One reviewer was academic advisor to Road to Health Ltd, and reports honorarium from AMGEN | Intellectual  Individual financial |
| Oates, 2019, Clinical Rehab | - One reviewer carried out the work in part fulfilment of the requirements of his Doctorate | ***Not classified according to framework*** |
| Poole, 2019, Cochrane | - One of the reviewers was an editor with Cochrane Airways. - One of the reviewers was a joint Co-ordinating Editor of Cochrane Airways | Intellectual |
| Robinson, 2019, Bone & Joint Journal | - The authors reported institutional grants from Stryker to the   University of Edinburgh, Edinburgh, United Kingdom and Glasgow Royal Infirmary Orthopaedic Research Unit, Glasgow, United Kingdom not related to this study.   - Three reviewers reported personal lecture payments from Stryker, not related to this study. - One reviewer reported personal consultancy fees from Corin,   Smith & Nephew, and Stryker, not related to this study | Institutional financial  Individual financial |
| Salam, 2019, J Hypertension | One reviewer received salary support in part from George Health Enterprises, the social enterprise arm of The George Institute, which has received investment for the development of fixed dose combination therapy containing statin, aspirin, and blood pressure lowering medications.  George Health Enterprises had submitted patents for low dose blood pressure combinations, on which one of the reviewers was listed as one of the inventors. None of the authors had a financial interest in these planned products. | ***Not classified according to framework*** (The George Institute = not for profit)  Intellectual |
| Shaheed, 2019, Br J Clin Pharmacol | Eight reviewers were involved in clinical trials research evaluating the use of metformin in dialysis patients. | Intellectual |
| Stausholm, 2019, BMJ Open | Two reviewers were post-presidents and former board members of World Association for Laser Therapy, a non-for-profit research organization from which did not receive funding, grants or fees. | Institutional advocatory |
| Stoner, 2019, Aging and Mental health | - One reviewer was offering Cognitive Stimulation Therapy (CST) training courses on a consultancy basis | Professional |
| Witt, 2019, Austr NZ J Psychiatry | - Three reviewers were involved in trials included in the systematic review | Intellectual |
| Wright, 2019, J Clin Psychiatry | - One reviewer was the author of a book/program used in one   included study.   - The same reviewer had equity interests in companies that develop and distribute the book and receives book royalties. - Another reviewer was an employee at SilverCloud Health - Another reviewer was a consultant to SilverCloud Health - Another reviewer received book royalties - Another reviewer was a consultant to several companies | Intellectual  Individual financial |
| Aslan, 2010, Diab Medicine | - One of the reviewers conducted one of the included trials | Intellectual |
| Eyding, 2010, BMJ | - One reviewer received remuneration from Boehringer Ingelheim and Lilly Pharma for three talks on depression guidelines within 3 years from publication of the systematic review - Two reviewers were involved in the development of the German Disease Management Guideline on Depression | Individual financial  Intellectual |
| Nkansah, 2010, Cochrane | - One reviewer was involved in a study that could be eligible for inclusion in a future update to the review. | Intellectual |
| Manheimer, 2010, Cochrane | - The review included trials in which some of the reviewers were involved. - One reviewer was using acupuncture in his clinical work. - One reviewer received travel reimbursements and honoraria. - Three reviewers received honoraria. | Intellectual  Professional  Individual financial |
| Walters, 2010, Cochrane | One reviewer was an investigator of an included study | Intellectual |
| Zammit, 2010, Cochrane | - Four of the reviewers were involved in a clinical trial on the efficacy of intra-articular hyaluronan for the treatment of first MPJ OA, at the same time. | Intellectual |

**Table S5.** Individual financial conflicts of interest (COI) as declared by authors of the included systematic reviews (SRs) for 2019 and 2010 by basic characteristics, systematic reviews from China excluded. Numbers in parentheses are percentages of the row total.

| **Characteristics** | SRs with ≥ 1 author declaring individual financial COI | SRs by authors declaring no COI, or only other than the individual financial type of COI | SRs missing information  (no COI statement) | **Total** |
| --- | --- | --- | --- | --- |
| **Systematic reviews 2019** |  |  |  |  |
| **Diagnosis** |  |  |  |  |
| T2DM | 26 (22) | 86 (72) | 7 (6) | **119** |
| COPD | 11 (24) | 31 (67) | 4 (9) | **46** |
| Hypertension | 3 (5) | 55 (93) | 1 (2) | **59** |
| Dementia | 7 (10) | 56 (81) | 6 (9) | **69** |
| Depression | 26 (38) | 36 (52) | 7 (10) | **69** |
| Osteoarthritis | 30 (29) | 66 (63) | 8 (8) | **104** |
| **Intervention** |  |  |  |  |
| Pharmacological | 58 (38) | 85 (56) | 8 (5) | **151** |
| Invasive procedures | 25 (35) | 41 (57) | 6 (8) | **72** |
| Psychological, social, nursing | 4 (4) | 75 (84) | 10 (11) | **89** |
| Physio-, occupational therapy | 3 (5) | 55 (87) | 5 (8) | **63** |
| Dietary | 8 (14) | 48 (84) | 1 (2) | **57** |
| Alternative, complementary | 0 | 9 (90) | 1 (10) | **10** |
| Multiple interventions | 5 (21) | 17 (71) | 2 (8) | **24** |
| **Country** |  |  |  |  |
| UK | 18 (26) | 48 (70) | 3 (4) | **69** |
| USA | 19 (27) | 44 (62) | 8 (11) | **71** |
| Australia | 10 (19) | 39 (75) | 3 (6) | **52** |
| Canada | 12 (38) | 19 (59) | 1 (3) | **32** |
| Other countries | 44 (18) | 180 (74) | 18 (7) | **242** |
| **Journal** |  |  |  |  |
| Cochrane | 11 (52) | 10 (48) | 0 | **21** |
| Other journals | 92 (21) | 320 (72) | 33 (7) | **445** |
| **Total for 2019** | **103 (22)** | **330* (71)** | **33 (7)** | **466** |
| **Systematic reviews 2010** |  |  |  |  |
| **Diagnosis** |  |  |  |  |
| T2DM | 5 (20) | 17 (68) | 3 (12) | **25** |
| COPD | 8 (30) | 15 (56) | 4 (15) | **27** |
| Hypertension | 4 (15) | 19 (73) | 3 (12) | **26** |
| Dementia | 4 (25) | 6 (38) | 6 (38) | **16** |
| Depression | 11 (32) | 17 (50) | 6 (18) | **34** |
| Osteoarthritis | 3 (14) | 10 (45) | 9 (41) | **22** |
| **Intervention** |  |  |  |  |
| Pharmacological | 21 (46) | 18 (39) | 7 (15) | **46** |
| Invasive procedures | 3 (21) | 6 (43) | 5 (36) | **14** |
| Psychological, social, nursing | 6 (16) | 28 (74) | 4 (10) | **38** |
| Physio-, occupational therapy | 2 (9) | 13 (59) | 7 (32) | **22** |
| Dietary | 2 (10) | 12 (60) | 6 (30) | **20** |
| Alternative, complementary | 0 | 3 (100) | 0 | **3** |
| Multiple interventions | 1 (14) | 4 (57) | 2 (29) | **7** |
| **Country** |  |  |  |  |
| UK | 6 (18) | 23 (68) | 5 (15) | **34** |
| USA | 7 (30) | 10 (43) | 6 (26) | **23** |
| Australia | 3 (18) | 9 (53) | 5 (29) | **17** |
| Canada | 6 (35) | 9 (53) | 2 (12) | **17** |
| Other countries | 13 (22) | 33 (56) | 13 (22) | **59** |
| **Journal** |  |  |  |  |
| Cochrane | 7 (25) | 20 (71) | 1 (4) | **28** |
| Other journals | 28 (23) | 64 (52) | 30 (25) | **122** |
| **Total for 2010** | **35 (23)** | **84** (56)** | **31 (21)** | **150** |

* Excluding Chinese systematic reviews, authors declare they have no COI (n=312) or declare they have COI, but it is not individual financial COI (n=121-103=18): 312+18=330.

** Excluding Chinese systematic reviews, authors declare they have no COI (n=80) or declare they have COI, but it is not individual financial COI (n=38-34=4): 80+4=84.

**Table S6.** Individual intellectual conflicts of interest (COI) as declared by authors of the included systematic reviews for 2019 and 2010 by basic characteristics. Numbers in parentheses are percentages of the row total.

| **Characteristics** | Systematic reviews  with ≥ 1 author declaring  individual intellectual COI | **Total** |
| --- | --- | --- |
| **Systematic reviews 2019** |  |  |
| **Diagnosis** |  |  |
| T2DM | 3 (2) | **161** |
| COPD | 1 (2) | **61** |
| Hypertension | 1 (1) | **77** |
| Dementia | 1 (1) | **83** |
| Depression | 5 (6) | **84** |
| Osteoarthritis | 4 (3) | **124** |
| **Intervention** |  |  |
| Pharmacological | 8 (4) | **189** |
| Invasive procedures | 0 | **86** |
| Psychological, social, nursing | 4 (4) | **101** |
| Physio-, occupational therapy | 3 (3) | **93** |
| Dietary | 0 | **70** |
| Alternative, complementary | 0 | **22** |
| Multiple interventions | 0 | **29** |
| **Country** |  |  |
| China | 0 | **124** |
| UK | 4 (6) | **69** |
| USA | 2 (3) | **71** |
| Australia | 3 (6) | **52** |
| Canada | 1 (3) | **32** |
| Other countries | 5 (2) | **242** |
| **Journal** |  |  |
| Cochrane | 5 (23) | **22** |
| Other journals | 10 (2) | **568** |
| **Total for 2019** | **15 (3)** | **590** |
| **Systematic reviews 2010** |  |  |
| **Diagnosis** |  |  |
| T2DM | 1 (4) | **25** |
| COPD | 1 (4) | **28** |
| Hypertension | 1 (3) | **29** |
| Dementia | 0 | **16** |
| Depression | 1 (3) | **35** |
| Osteoarthritis | 2 (9) | **23** |
| **Intervention** |  |  |
| Pharmacological | 1 (2) | **50** |
| Invasive procedures | 0 | **15** |
| Psychological, social, nursing | 3 (8) | **38** |
| Physio-, occupational therapy | 1 (4) | **23** |
| Dietary | 0 | **20** |
| Alternative, complementary | 0 | **3** |
| Multiple interventions | 1 (14) | **7** |
| **Country** |  |  |
| China | 0 | **6** |
| UK | 1 (3) | **34** |
| USA | 2 (9) | **23** |
| Australia | 2 (12) | **17** |
| Canada | 0 | **17** |
| Other countries | 1 (2) | **59** |
| **Journal** |  |  |
| Cochrane | 4 (14) | **29** |
| Other journals | 2 (2) | **127** |
| **Total for 2010** | **6 (4)** | **156** |

**Table S7.** Presence of a statement on funding by basic characteristics in the included systematic reviews for 2019 and 2010. Numbers in parentheses are row percentages.

| **Characteristics** | Systematic reviews reporting statement on funding | Systematic reviews missing statement on funding | **Total** |
| --- | --- | --- | --- |
| **Systematic reviews 2019** |  |  |  |
| **Diagnosis** |  |  |  |
| T2DM | 135 (84) | 26 (16) | **161** |
| COPD | 54 (89) | 7 (11) | **61** |
| Hypertension | 49 (64) | 28 (36) | **77** |
| Dementia | 70 (84) | 13 (16) | **83** |
| Depression | 71 (85) | 13 (15) | **84** |
| Osteoarthritis | 96 (77) | 28 (23) | **124** |
| **Intervention** |  |  |  |
| Pharmacological | 145 (77) | 44 (23) | **189** |
| Invasive procedures | 63 (73) | 23 (27) | **86** |
| Psychological, social, nursing | 86 (85) | 15 (15) | **101** |
| Physio-, occupational therapy | 78 (84) | 15 (16) | **93** |
| Dietary | 60 (86) | 10 (14) | **70** |
| Alternative, complementary | 17 (77) | 5 (23) | **22** |
| Multiple interventions | 26 (90) | 3 (10) | **29** |
| **Country** |  |  |  |
| China | 108 (87) | 16 (13) | **124** |
| UK | 62 (90) | 7 (10) | **69** |
| USA | 53 (75) | 18 (25) | **71** |
| Australia | 46 (88) | 6 (12) | **52** |
| Canada | 20 (63) | 12 (38) | **32** |
| Other countries | 186 (77) | 56 (23) | **242** |
| **Journal** |  |  |  |
| Cochrane | 21 (95) | 1 (5) | **22** |
| Other journals | 454 (80) | 114 (20) | **568** |
| **Total for 2019** | 475 (81) | 115 (19) | **590** |
| **Systematic reviews 2010** |  |  |  |
| **Diagnosis** |  |  |  |
| T2DM | 12 (48) | 13 (52) | **25** |
| COPD | 20 (71) | 8 (29) | **28** |
| Hypertension | 22 (76) | 7 (24) | **29** |
| Dementia | 8 (50) | 8 (50) | **16** |
| Depression | 23 (66) | 12 (34) | **35** |
| Osteoarthritis | 16 (70) | 7 (30) | **23** |
| **Intervention** |  |  |  |
| Pharmacological | 31 (62) | 19 (38) | **50** |
| Invasive procedures | 8 (53) | 7 (47) | **15** |
| Psychological, social, nursing | 24 (63) | 14 (37) | **38** |
| Physio-, occupational therapy | 19 (83) | 4 (17) | **23** |
| Dietary | 10 (50) | 10 (50) | **20** |
| Alternative, complementary | 3 (100) | 0 | **3** |
| Multiple interventions | 6 (86) | 1 (14) | **7** |
| **Country** |  |  |  |
| China | 4 (67) | 2 (33) | **6** |
| UK | 21 (62) | 13 (38) | **34** |
| USA | 15 (65) | 8 (35) | **23** |
| Australia | 10 (59) | 7 (41) | **17** |
| Canada | 15 (88) | 2 (12) | **17** |
| Other countries | 36 (61) | 23 (39) | **59** |
| **Journal** |  |  |  |
| Cochrane | 26 (90) | 3 (10) | **29** |
| Other journals | 75 (59) | 52 (41) | **127** |
| **Total for 2010** | 101 (65) | 55 (35) | **156** |

**Table S8.** Funding from industry as declared in the included systematic reviews for 2019 and 2010 by basic characteristics. Numbers in parentheses are row percentages.

| **Characteristics** | Systematic reviews declaring funding from industry | Systematic reviews declaring no funding from industry or only non-industry funding | Systematic reviews missing information  (no funding statement) | **Total** |
| --- | --- | --- | --- | --- |
| **Systematic reviews 2019** |  |  |  |  |
| **Diagnosis** |  |  |  |  |
| T2DM | 7 (4) | 128 (80) | 26 (16) | **161** |
| COPD | 5 (8) | 49 (80) | 7 (11) | **61** |
| Hypertension | 1 (1) | 48 (62) | 28 (36) | **77** |
| Dementia | 0 | 70 (84) | 13 (16) | **83** |
| Depression | 0 | 71 (85) | 13 (15) | **84** |
| Osteoarthritis | 7 (6) | 89 (72) | 28 (23) | **124** |
| **Intervention** |  |  |  |  |
| Pharmacological | 13 (7) | 132 (70) | 44 (23) | **189** |
| Invasive procedures | 2 (2) | 61 (71) | 23 (27) | **86** |
| Psychological, social, nursing | 1 (1) | 85 (84) | 15 (15) | **101** |
| Physio-, occupational therapy | 1 (1) | 77 (83) | 15 (16) | **93** |
| Dietary | 2 (3) | 58 (83) | 10 (14) | **70** |
| Alternative, complementary | 0 | 17 (77) | 5 (23) | **22** |
| Multiple interventions | 1 (3) | 25 (86) | 3 (10) | **29** |
| **Country** |  |  |  |  |
| China | 1 (1) | 107 (86) | 16 (13) | **124** |
| UK | 2 (3) | 60 (87) | 7 (10) | **69** |
| USA | 6 (8) | 47 (66) | 18 (25) | **71** |
| Australia | 2 (4) | 44 (85) | 6 (12) | **52** |
| Canada | 2 (6) | 18 (56) | 12 (38) | **32** |
| Other countries | 7 (3) | 179 (74) | 56 (23) | **242** |
| **Journal** |  |  |  |  |
| Cochrane | 0 | 21 (95) | 1 (5) | **22** |
| Other journals | 20 (4) | 434 (76) | 114 (20) | **568** |
| **Total for 2019** | **20 (3)** | **455* (77)** | **115 (19)** | **590** |
| **Systematic reviews 2010** |  |  |  |  |
| **Diagnosis** |  |  |  |  |
| T2DM | 2 (8) | 10 (40) | 13 (52) | **25** |
| COPD | 2 (7) | 18 (64) | 8 (29) | **28** |
| Hypertension | 0 | 22 (76) | 7 (24) | **29** |
| Dementia | 2 (13) | 6 (38) | 8 (50) | **16** |
| Depression | 3 (9) | 20 (57) | 12 (34) | **35** |
| Osteoarthritis | 1 (4) | 15 (65) | 7 (30) | **23** |
| **Intervention** |  |  |  |  |
| Pharmacological | 6 (12) | 25 (50) | 19 (38) | **50** |
| Invasive procedures | 0 | 8 (53) | 7 (47) | **15** |
| Psychological, social, nursing | 0 | 24 (63) | 14 (37) | **38** |
| Physio-, occupational therapy | 1 (4) | 18 (78) | 4 (17) | **23** |
| Dietary | 2 (10) | 8 (40) | 10 (50) | **20** |
| Alternative, complementary | 0 | 3 (100) | 0 | **3** |
| Multiple interventions | 1 (14) | 5 (71) | 1 (14) | **7** |
| **Country** |  |  |  |  |
| China | 0 | 4 (67) | 2 (33) | **6** |
| UK | 1 (3) | 20 (59) | 13 (38) | **34** |
| USA | 3 (13) | 12 (52) | 8 (35) | **23** |
| Australia | 0 | 10 (59) | 7 (41) | **17** |
| Canada | 2 (12) | 13 (76) | 2 (12) | **17** |
| Other countries | 4 (7) | 32 (54) | 23 (39) | **59** |
| **Journal** |  |  |  |  |
| Cochrane | 0 | 26 (90) | 3 (10) | **29** |
| Other journals | 10 (8) | 65 (51) | 52 (41) | **127** |
| **Total for 2010** | **10 (6)** | **91** (58)** | **55 (35)** | **156** |

* Authors declare there was no funding (n=167) or declare there was funding but not from industry (n=308-20=288): 167+288=455.

** Authors declare there was no funding (n=14) or declare there was funding but not form industry (n=87-10=77): 14+77=91.
